# Notes and News

**Published:** 1894-10-06

**Authors:** 


### Notes and News.

Collected and Communicated.

A legacy of 15,000 francs from Madam Halff is to be devoted to the endowment of a bed for an aged male patient at the Hospital Rothschild at Paris.

Sir George Murray Humphry, M.D., Professor of Surgery, has tendered his resignation of the office of Senior Surgeon to Addenbrooke's Hospital, Cambridge, with which institution he has been connected for over half a century, and at the same time forwarded to the governors a cheque for £500 for the purpose of improving the out-patient accommodation in the surgical department. At the last quarterly court, held on Monday, a resolution was passed appointing him consulting surgeon to the hospital, subject to his acceptance of the post. A cordial vote of thanks was also given to Sir George M. Humphry for his munificent donation of £500 for the purpose of improving the out-patient accommodation in the surgical department; and it was resolved that a committee of the medical and surgical officers and the select governors should be appointed to consider the best way of carrying out the improvement, with power to obtain plans and estimates to be laid before the next quarterly court.

We are informed that the authorities of the Great Northern Central Hospital announce that from October 1st they will open their pay-wards to the public. These wards will be conducted upon somewhat novel lines, and the experiment will be of interest to all friends of medical charities. The wards are intended solely for patients of limited means who are unable to afford the necessary medical or surgical treatment at their own homes, but who are willing to contribute something towards the cost of their maintenance when in the hospital. The charges will be £2 2s. per week, or less, the amount of the payment being fixed by the committee after consideration of the circumstances of each case. Particular care will be taken to restrict the benefits of the important branch of the charity to the most suitable and deserving cases. The medical staff have undertaken to attend the patients gratuitously, on the same conditions as the patients in the free wards.

The hospital accommodation of Belfast is to be extended by the building of a new Mater Infirmorum Hospital. It will be established for the relief of the sick and dying poor, without distinction of creed. A large public meeting in favour of the movement was held on September 24th, at which the Bishop of Down and Connor presided.

The London Temperance Hospital comes of age on the 8th of the present month. To celebrate its majority, the Board of Management will hold a reception on that date, when all visitors will be welcome after two o'clock to view the hospital. Tickets of admission can be obtained from the secretary at the hospital on written application. We have heard much admiration expressed at the bright wards of the institution, and some of the sanitary arrangements are quite model, and we advise all who can do so to take advantage of the invitation of the committee.

The Annual Congress of the Sanitary Institute, held this year in Liverpool, has been both successful and interesting. Sir F. S. Powell, M.P., delivered the Presidential address, in which he surveyed the whole field of subjects under consideration of the Congress. His remarks upon the subject of vaccination were wise and temperate, and he expressed himself hopeful in respect of the non-increase of insanity. It was pleasant to hear him so emphatic on the point that the smoke nuisance must be abated. The range of subjects was a wide one, but they were all treated in a telling manner. The various sections were ably presided over; the subjects, being of general interest, secured good audiences.

Leicester is really an unfortunate place. Not only have an undue proportion of epidemic visitations fallen to its share, but now it is visited with a water famine. The absence of sun during the summer causes us to regard a drought with surprise. Nevertheless Leicester is now put to great expense and its sanitary condition seriously endangered. The sources upon which the town is now depending are far from satisfactory, and even when the whole amount is available the supply will be most limited. The Midland Railway Company are employing 500 men to lay down pipes from a distance of five miles from Leicester that they may be able to supply their engines.

An interesting article on the epidemic skin disease, which has lately visited several London workhouse infirmaries, appears in a medical contemporary. As we stated, an enquiry is being made, and is not yet complete. In the enquiry special attention is being paid to the inference that the epidemic was in some way connected with the milk consumed by those attacked, two of the infirmaries being supplied from the same source. Those who have studied this special form of skin disease, are not of the opinion that there is of necessity any connection between the milk supply and the outbreak. The disease is peculiarly infectious, attacks the old and feeble, and by its liability to show itself in workhouse infirmaries is detected with great facility by the medical officers of those institutions. It must not be supposed, however, that it is peculiar to union inmates, as it is a recognised visitant in many hospitals.

